# Supplementary material for: Sustainable Treatment and Resource Recovery of Anion Exchange Spent Brine by Pilot-Scale Electrodialysis and Ultrafiltration
Source: Membranes (Basel). 2022 Feb 27;12(3):273. doi: 10.3390/membranes12030273 (PMC8953537; doi:10.3390/membranes12030273)
Supplement: Supplementary file 1 [file membranes-12-00273-s001.zip › membranes-1607892-supplementary.pdf]

membranes

Supplementary Materials: Sustainable treatment and resource recovery of anion exchange spent brine by pilot-scale electrodialysis and ultrafiltration

Hongfang Sun [1], Daoxu Zhu [1], Peng Shi [1,2], Wenxiang Ji [1], Xun Cao [2], Shi Cheng [1], Yufeng Lou [4] and Aimin Li [1,2,3,*]

[1] State Key Laboratory of Pollution Control and Resources Reuse, School of the Environment, Nanjing University, Nanjing 210023, China; shf4514356@163.com (H.S.); 18851777819@163.com (D.Z.); shipeng@nju.edu.cn (P.S.); jwx1996_nju@163.com (W.J.); shicheng_nju@163.com (S.C.)
[2] Nanjing University & Yancheng Academy of Environmental Protection Technology and Engineering, Yancheng 224000, China; caoxun890310@163.com
[3] Quanzhou Institute for Environmental Protection Industry, Nanjing University, Quanzhou 362008, China
[4] Key Laboratory of Charged Polymeric Membrane Materials of Shandong Province, Shandong Tianwei Membrane Technology Co., Ltd., 13173 Yuqing East Street, Weifang, China; yf_lou@163.com
* Correspondence: liaimingroup@nju.edu.cn

1. Detailed experimental conditions of two-stage pilot-scale ED

The separation performance of the two-stage pilot-scale ED (Figure S1) for the anion exchange spent brine was investigated with TWED-32-100 electrodialysis pilot plant (Shandong Tianwei Membrane Technology co. Ltd.) with 100 pairs of cells composed of cationic and anionic membranes (TWEDC1/TWEDA1 membrane pair, 64 m^2 membrane area). The processing scale of ED pilot-scale equipment is 5 tons anion exchange spent brine per day. The pilot-scale electrodialysis of AIX spent brine was carried out under potentiostatic condition at 70 V. And the batch operating mode was used. In the pilot-scale first-stage ED process, the anion exchange spent brine (feed solution, 180 L, COD_{Mn} = 1200-4000 mg/L, NaCl content is 8-10% w/w, pH ≈ 8), tap water (60 L) and 3% (w/w) NaCl solution (90 L) were respectively used as first-stage dilute solution, first-stage concentrate solution and electrode solution. The flow rates of electrode chamber, dilute chamber and concentrate chamber were 1300 L/h, 2.5 m^3/h and 2.5 m^3/h, respectively. DC power supplied the constant voltage of 70 V and the pressure was controlled at 0.01~0.02 MPa in the pilot-scale ED process. The dilute solution of first-stage ED was further desalinated to minimize salt content by the second-stage ED. In the pilot-scale second-stage ED process, the first-stage dilute solution of ED (feed solution), tap water and 3% (w/w) NaCl solution were respectively used as second-stage dilute solution, second-stage concentrate solution and electrode solution. The volume ratio of initial second-stage concentrate solution to second-stage dilute solution was 1:3.

2. SEC-DAD-FLD-OCD systems

The size exclusion chromatography (SEC)-diode array detector (DAD)-fluorescence detector (FLD)-organic carbon detector (OCD) analyses [1, 2] utilizing an Agilent 1260 HPLC System equipped with a diode array detector (DAD, Agilent 1290 Infinity II G7117B), a fluorescence detector (FLD, Agilent 1260 Infinity II G1721B), an online organic carbon detector (Turbo Sievers® M9SEC TOC Analyzer, Suez) and installed a TSK HW-50S size exclusion column (20 mm × 250 mm, ordered from DOC-Labor, Karlsruhe, Germany) were used to measure the characteristics of anion exchange spent brine, second-

stage dilute solution, first-stage concentrate solution and second-stage concentrate solution in order to investigate the transport of organic matters through the ion exchange membranes (IEMs) in the two-stage pilot-scale ED process. DAD chromatogram with UV absorbance scans from 200 nm to 300 nm, while FLD chromatogram with emission scans from 300 to 480 nm with excitation at 230 nm. The injection volume was 500 μL. The mobile phase was phosphate buffer (1.5 g/L $Na_2HPO_4{\cdot}2H_2O$ + 2.5 g/L KH_2PO_4) with 1 mL/min flow rate to suppress ionic interaction [1].

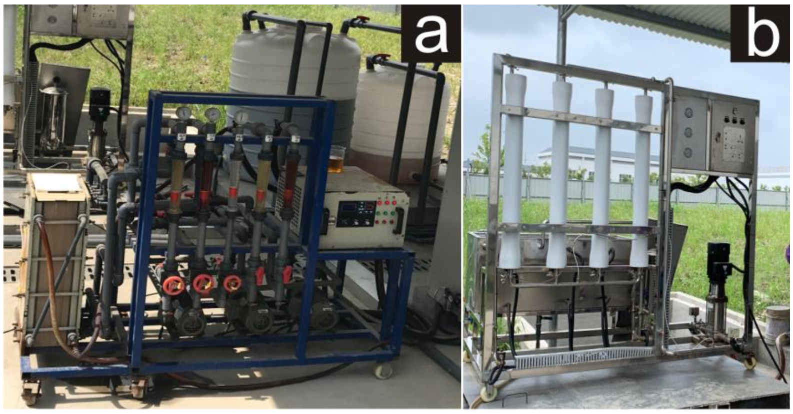

Figure S1. Photos of ED+UF pilot test on site. (a) ED pilot-scale equipment, (b) UF pilot-scale equipment.

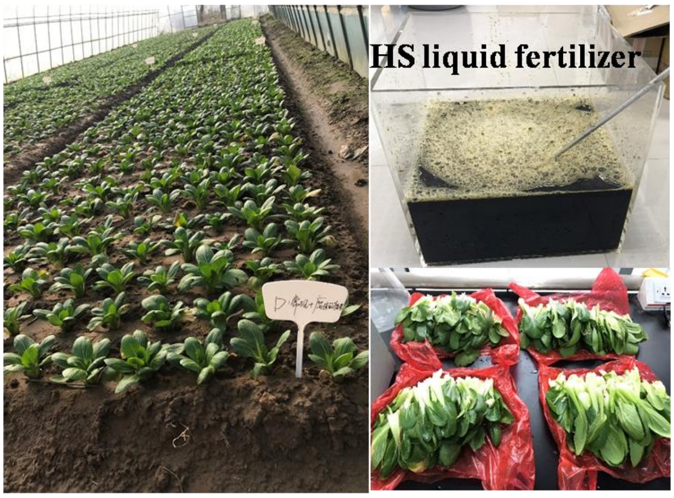

Figure S2. Photos of the pilot test on cultivation of green vegetables.

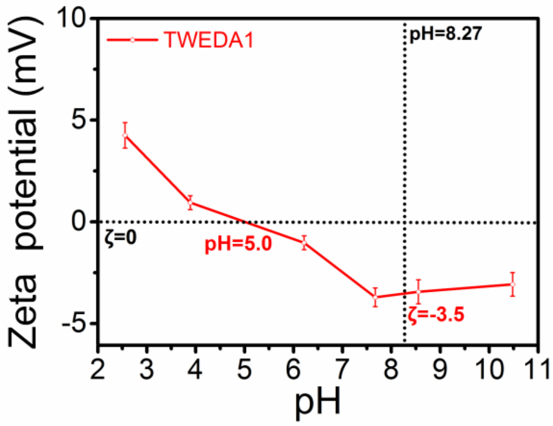

Figure S3. Zeta potential of TWEDA1 anion exchange membrane versus pH value.

Table S1. Properties of TWEDC1/TWEDA1 membranes[a].

Membrane	[b]Type and materials	[b]Reinforcement	[b]IEC/ meq·g^{-1}	Water content (%)	Fixed charge concentration (mol/L)	Electric resistance /Ω·cm^{-2}	Permselectivity (%)	Contact angle (Degree)
TWEDC1	Homogeneous sulphonated PPO cation exchanger	Polyester [3]	0.9-1.1	15	6-7.3	≤3.0	≥96	60.30
TWEDA1	co-(DMC-DMAMA-DVB) [4]	Nylon 6,6 [4]	0.9-1.1	15	6-7.3	≤2.5	≥97	66.93

[a] These data were provided by manufacturer, literatures and experiments.
[b] IEC = ion exchange capacity, DVB = divinylbenzene, PPO = polymer poly(2,6-dimethyl-1,4-phenylene oxide), DMC = dimethyl carbonate.

Table S2. Effect of influent flow on concentration efficiency in pilot UF.

Influent flow (L/min) / Parameter	16.9	19.1	24.4	25.4	27.9
Volume concentration factor *CF*	3.4	3.5	2.1	2.4	2.2
Membrane permeation flux *J* (L•m^{-2}•h^{-1})	22.2	20.4	28.3	34.2	30.0
Rejection coefficient *R* (%)	89.8	97.8	99.3	98.2	93.3
COD_{Mn} of two-stage dilute solution in ED pilot (mg/L)	5393.8	4754.8	4971.4	4969.8	5563.4
COD_{Mn} of retentate solution in UF pilot (mg/L)	13722.6	5292.4	9878.9	3053.3	7293.3
COD_{Mn} of permeate solution in UF pilot (mg/L)	550	103.7	87.1	32.7	371.9

Table S3. Composition of conventional fertilizer and HS liquid fertilizer.

Conventional fertilizer		HS fertilizer	
**Urea (kg)**	**7.76**	HS (g/L)	> 30
Calcium superphosphate(kg)	10.0	N (g/L)	78.3
K_2SO_4 (kg)	8.0	P_2O_5 (g/L)	34.8
-	-	K_2O (g/L)	87.0

Table S4. Running cost of sustainable treatment of AIX spent brine.

Treatment processes	Operating cost (Yuan/ton)
ED pilot	20
UF pilot	0.67
NaCl recovery	-31.5[a]
Total cost	-10.83[a]

[a]Negative sign indicates profit.

Reference.

1. Cai, M.-H.; Wu, Y.-P.; Ji, W.-X.; Han, Y.-Z.; Li, Y.; Wu, J.-C.; Shuang, C.-D.; Korshin, G.V.; Li, A.-M.; Li, W.-T. Characterizing property and treatability of dissolved effluent organic matter using size exclusion chromatography with an array of absorbance, fluorescence, organic nitrogen and organic carbon detectors. *Chemosphere* **2020**, *243*, 125321.
2. Huber, S.A.; Balz, A.; Abert, M.; Pronk, W. Characterisation of aquatic humic and non-humic matter with size-exclusion chromatography-organic carbon detection-organic nitrogen detection (LC-OCD-OND). *Water Res.* **2011**, *45*, 879–885.
3. Xu, T.W.; Li, Y.; Wu, L.; Yang, W.H. A simple evaluation of microstructure and transport parameters of ion-exchange membranes from conductivity measurements. *Sep. Purif. Technol.* **2008**, *60*, 73–80.
4. Wei, B.; Feng, J.; Chen, C.; Zhong, S.; Liao, S.; Yu, Y.; Li, X. Highly permselective tadpole-type ionic anion exchange membranes for electrodialysis desalination. *J. Membr. Sci.* **2020**, *600*, 117861.

.
